# Peripheral microbial metabolites as indicators of gut microbiome disruption: systematic review and meta-analysis

**DOI:** 10.1128/mbio.01927-26

**Published:** 2026-10-07

**Authors:** Taylor Kain, Eric Armstrong, Bryan Coburn

**Affiliations:** 1 Institute of Medical Science, Department of Medicine, University of Toronto 7938, Toronto, Ontario, Canada; 2 Toronto General Hospital Research Institute, University Health Network 7989, Toronto, Ontario, Canada; Universite de Geneve, Geneva, Switzerland

**Keywords:** gut microbiome, metabolomics, circulating metabolites, bile acids, short-chain fatty acids, anaerobe depletion, biomarkers, systematic review, meta-analysis

## Abstract

Gut microbiome disruption is often characterized by the loss of obligately anaerobic bacteria, which may lead to altered production of microbial metabolites that can be detected peripherally. The application of widely used sequencing-based microbiome analyses to clinical settings is limited by cost, turnaround time, and challenges with patients with very low stool output. Since some products of strictly bacterial metabolism are detectable in blood, peripheral metabolites may provide a rapid and scalable indicator of gut microbiome composition and function. We performed a systematic review and meta-analysis of studies reporting circulating microbial metabolites and gut microbiome composition to evaluate whether peripheral microbial metabolites could identify gut microbiome perturbation. Candidate metabolites were identified systematically across an independent set of studies reporting metabolite-microbiome associations, enabling the assessment of reproducibility across disease states and cohorts. We performed a meta-analysis of 19 human cohorts comprising 3,242 participants with paired blood metabolite and stool microbiome data. Anaerobe depletion (obligate anaerobe relative abundance <0.70) was associated with decreased plasma concentration of products of anaerobic microbial metabolism. Combinations of metabolites distinguished individuals with anaerobe-depleted microbiomes from those without. Circulating metabolite levels distinguished between cases and controls with similar performance as gut microbiome composition across a range of health/disease states and changed markedly within patients experiencing gut anaerobe depletion after antibiotic exposure. Circulating microbial metabolites are potentially informative indicators of gut microbiome disruption and may serve as a rapid and scalable method for patient stratification in clinical trials or acute care settings.

**IMPORTANCE:**

Circulating microbial metabolites represent a practical and scalable approach to detecting significant gut microbiome disruption, particularly the loss of obligate anaerobes. Our findings suggest that metabolites capture compositional consequences of microbiome collapse, with performance comparable to direct microbiome profiling in distinguishing disease states. Enabling diagnostic enrichment and real-time monitoring of microbiome injury (e.g., during antibiotic use or critical illness) has potential implications for both clinical care and research, including the selection of patients for investigation of microbiome-targeted therapies. With further validation, circulating metabolites could provide an accessible surrogate for gut microbiome composition in settings where sequencing is impractical.

## INTRODUCTION

In health, the gut microbiome is composed primarily of obligate anaerobes (1–3) and plays important roles in the regulation of host immune homeostasis, energy metabolism, and resistance to pathogen colonization (3–5). Gut microbiome disruption and anaerobe depletion are often observed in acute illness and have been implicated in disease risk, severity, and treatment responsiveness (6–9). Real-time identification of patients with disrupted microbiomes may enable timely interventions to restore gut anaerobes and improve clinical outcomes (10–15).

The primary method of analyzing the microbiome in humans is either 16S rRNA gene sequencing or shotgun metagenomics (16). Sequencing is slower and more costly than conventional laboratory testing, impeding the introduction of microbiome profiling into clinical settings such as acute and critical illness. However, gut microorganisms produce an array of metabolites (17–19) that enter systemic circulation and can be measured in the blood (20, 21). Measurement of specific analytes in plasma or serum is routinely integrated into clinical practice, with rapid, scalable, and inexpensive workflows for sample collection and analysis.

We hypothesized that circulating levels of metabolites known to be either primarily produced or utilized by microbes would correlate with gut microbiome disruption and identify individuals with substantial loss of gut obligate anaerobes. We performed a systematic review and meta-analysis to evaluate the consistency of these associations across diverse disease states.

## MATERIALS AND METHODS

### Literature search

**Fig 1:**
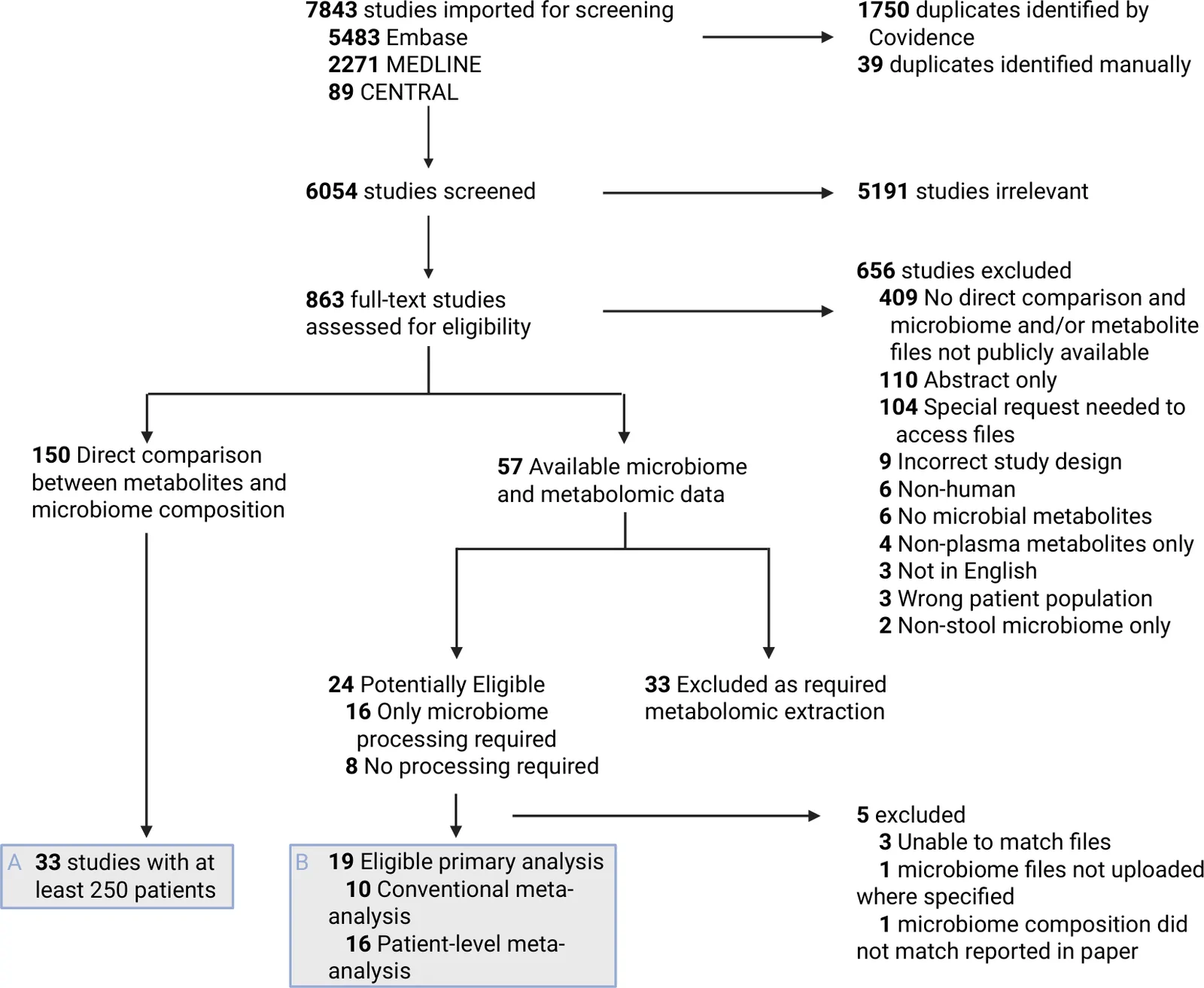
PRISMA diagram of studies screened and included in the systematic review. Box A represents studies included in the primary analysis, while bBox B represents independent studies used to generate a list of candidate metabolites used in the meta-analyses.

To identify eligible studies, we developed a protocol in accordance with the Preferred Reporting Items for Systematic Reviews and Meta-Analyses (PRISMA) checklist (22), which was registered with the International Prospective Register of Systematic Reviews (reference no. CRD42024504053). We searched for relevant studies across three databases (MEDLINE, Embase, and Cochrane) in February 2024 (full search strategy is provided in Table S1). We included articles that were only in English and excluded papers prior to the year 2000. We imported all abstracts into Covidence (Veritas Health Innovation) (23) for review. We included studies that either directly compared circulating (i.e., serum/plasma) metabolite concentrations with gut microbiome composition or studies where both peripheral metabolite and gut microbiome data were publicly and easily available to be secondarily analyzed. Any studies that required special access or primary analysis of raw mass spectrometry data were excluded. We excluded all animal studies as well as any studies of only non-peripheral metabolite levels or non-intestinal microbiomes. Two authors (T.K. and E.A.) independently screened abstracts and then full texts. We constructed a PRISMA diagram of included studies (Fig. 1); studies in Box A were used to generate a list of candidate metabolites, while studies in Box B were used in the primary analysis.

In total, 150 studies met the inclusion criteria but did not have publicly available metabolite or microbiome data; instead, they made direct comparisons between the metabolite and gut microbiome features (box A). Metabolites that had associations with groups of bacteria of interest (e.g., families of obligate anaerobes and potential pathogens) or with global microbiome metrics (e.g., alpha-diversity) in multiple studies scored more points and were more likely to be included. Further details of metabolite selection are described in Supplemental methods. From this analysis, 22 candidate microbial-host co-metabolites (subsequently referred to as microbial metabolites) were selected, and we hypothesized that these metabolites were the most likely to correlate with gut microbiome composition. Details of metabolite classification are provided in Table S3. Metabolites with modifications from human metabolism (i.e*.,* addition of a sulfate group by the liver) were grouped together if only one metabolite was reported within a study.

### Metadata extraction

For each included study, two reviewers (T.K. and E.A.) concurrently extracted metadata including publication year, location of the study, main disease state(s) of study participants, dates of subject enrollment, study design, baseline characteristics (e.g., age, sex, race, etc., where available), study interventions, and relevant clinical outcomes (if applicable). Quality assessment and risk-of-bias tools were not used, as analysis was done with primary data.

### Microbiome data extraction

16S rRNA and metagenomic sequencing data were downloaded in FASTQ format from public repositories and were processed by performing quality filtering and denoising of reads. Adapters were trimmed, and human reads were removed for metagenomic sequencing. Amplicon sequence variants (ASVs) were taxonomically annotated and collapsed to the genus level (24). Samples were rarefied to 5,000 reads (16S) and 500,000 reads (metagenomics), and all samples below these thresholds were excluded. Bacterial genera were classified as anaerobes, non-anaerobes, or unclassified based on descriptions in *Bergey’s Manual of Systematic Bacteriology* (25) and/or supporting literature.

### Metabolite data extraction

Metabolite levels were extracted from public data repositories or the study’s Supplemental material. We did not reprocess metabolomic data and used metabolite annotations provided from primary studies. Details of annotations in the primary studies are provided in File S3. Most studies reported metabolite levels as batch-normalized and unitless values, and the true lower limit of detection (LOD) for each metabolite was therefore not reported. If metabolite values were reported as below LOD, then they were imputed as half the lowest metabolite value reported for that metabolite in that study (26, 27). To manage unitless values on different scales, we employed two techniques: (i) conventional study-level meta-analysis comparing relationships between gut microbiome composition and metabolite levels and (ii) patient-level meta-analysis after normalization with a reference metabolite.

### Data visualization and conventional meta-analysis methods

Several studies had longitudinal samples collected. To decrease repeated measures bias, only one time point per patient was included, except in the longitudinal analysis. The middle time point was selected for each of these studies to increase the inclusion of more disrupted gut microbiomes in the analysis.

To predict gut microbiome disruption, an anaerobe relative abundance (RA) cutoff of 0.70 was used, approximating the lowest 10% of healthy individuals based on published data (3, 28), and samples were classified as “normal/high” or “low” based on this cutoff. Notably, this cutoff closely approximated the 10th percentile of anaerobe RA within the meta-analysis population (Fig. S1). Further details in Supplemental methods.

To examine the relationship between individual metabolites and anaerobe RA, metabolite values were normalized within each study to account for inter-study differences. Values were *z*-score-normalized within each study, and the mean z-scores were calculated per study and metabolite. A subset of metabolites, including deoxycholate (DCA), ursodeoxycholate (UDCA), indole-3-propionate (I3P), hippurate, phenylalanine, p-cresol sulfate (PCS), p-cresol glucuronide (PCG), and phenylacetylglutamine (PAG), was analyzed across studies with sufficient representation. For each study, the proportion of samples in each quadrant of anaerobe RA (high/low) versus metabolite level (above/below mean) was calculated.

### Patient-level meta-analysis methods

To combine patient-level data, we normalized metabolites using a reference metabolite level. Similar strategies have been utilized elsewhere in the literature (29). To select the reference metabolite, we calculated the average coefficient of variation of all metabolites across all studies and selected the analyte with the lowest average variance that was also represented in all studies (histidine). Further details are in the Supplemental methods.

### Case-control methods

All studies from the conventional meta-analysis that reported disease cases and controls were selected if data were available for more than one microbial metabolite. Random-forest classification models were used to classify cases from controls using (i) gut microbiome data in the form of genus-level RA and (ii) levels of all candidate metabolites. Only participants with paired microbiome and metabolite data were included. Prior to the generation of random forest models, data sets were split 70/30 into test/train sets. Random forest models were generated, with 10-fold cross-validation repeated three times. The area under the curve (AUC) for each model was obtained. Clustering of gut microbiome data was done with principal coordinate analysis (PCoA) using Bray-Curtis dissimilarities generated from genus-level gut microbiome data. Clustering of metabolite data was done with principal component analysis (PCA).

### Longitudinal analysis methods

For Rashidi et al., metabolite abundances were log10(*x* + 1) transformed due to skew. Patients were categorized as “dominated” or “never dominated” based on *Enterococcus* domination at any time point during the study. Domination was defined as *Enterococcus* RA >30% and at least double the next most abundant taxon (30).

For Tanes et al., RA of anaerobic bacteria, Shannon diversity, and reported metabolite concentrations (indole-3-propionate, imidazole propionate, and phenylalanine) were binned in 5-day intervals (0–5, 6–9, and 10–15 days) relative to study initiation. Longitudinal trends were visualized with thicker locally estimated scatterplot smoothing curves with 95% confidence intervals (31).

### Statistical analysis

**Fig 2:**
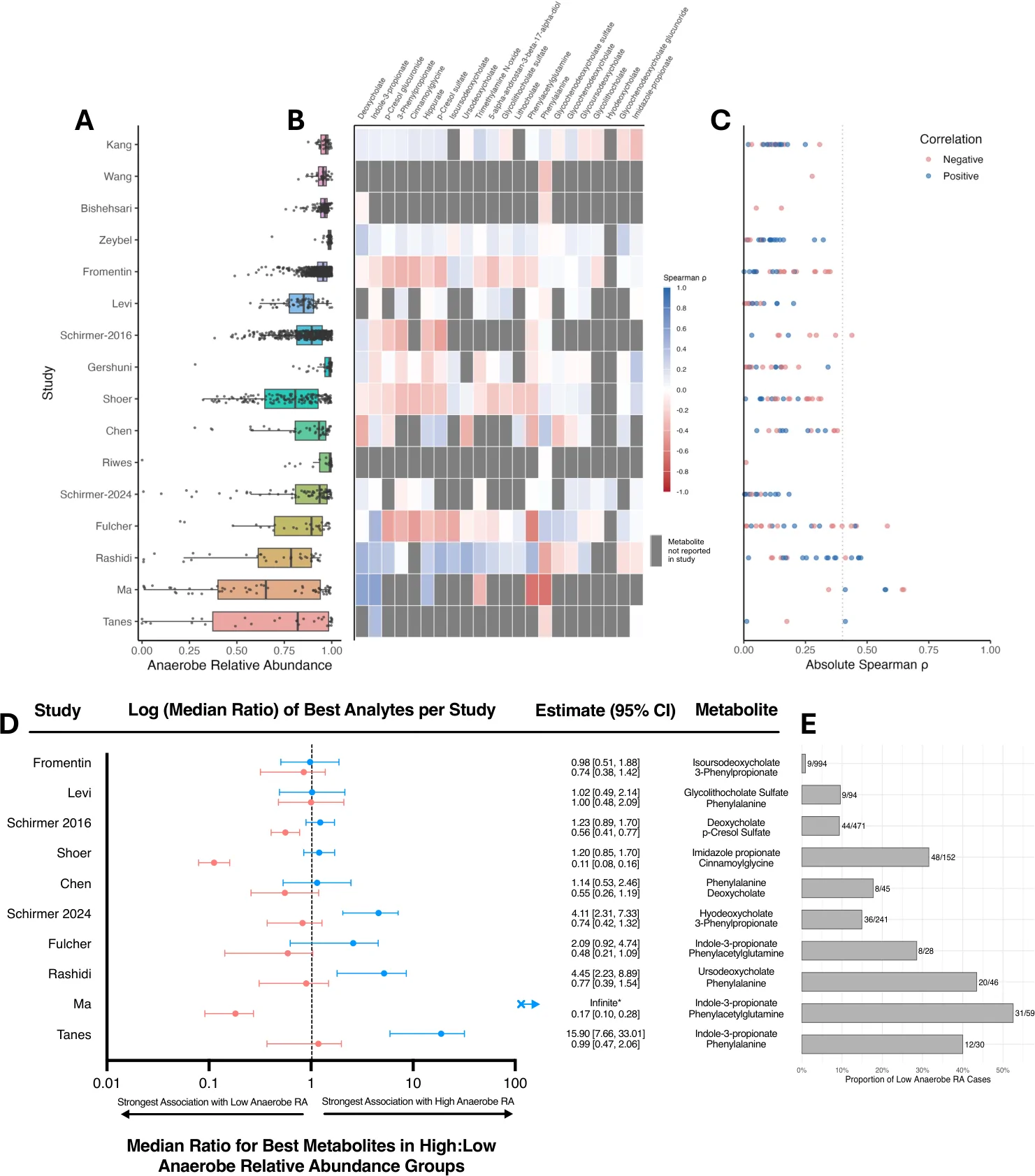
Correlations of metabolites and anaerobe RA and conventional meta-analysis of the best-performing analytes. (**A**) Anaerobe RA by study in order of increasing variance. (**B**) A heatmap showing Spearman correlations between each metabolite and anaerobe RA. Gray boxes indicate that the metabolite was not reported in that study. (**C**) Absolute Spearman correlations between peripheral metabolites and stool anaerobe RA. Dotted line shows Spearman >0.4. (**D**) Forest plot showing the median ratio between anaerobe RA >0.70 or <0.70 most positively and negatively correlated metabolites in each study. Confidence intervals were calculated using bootstrapping. (**E**) Proportion of participants with anaerobe RA <0.70 in each study. *Indole-3-propionate in Ma et al. had an infinite median ratio as all patients in the low anaerobe RA group had a level of 0.

**Fig 3:**
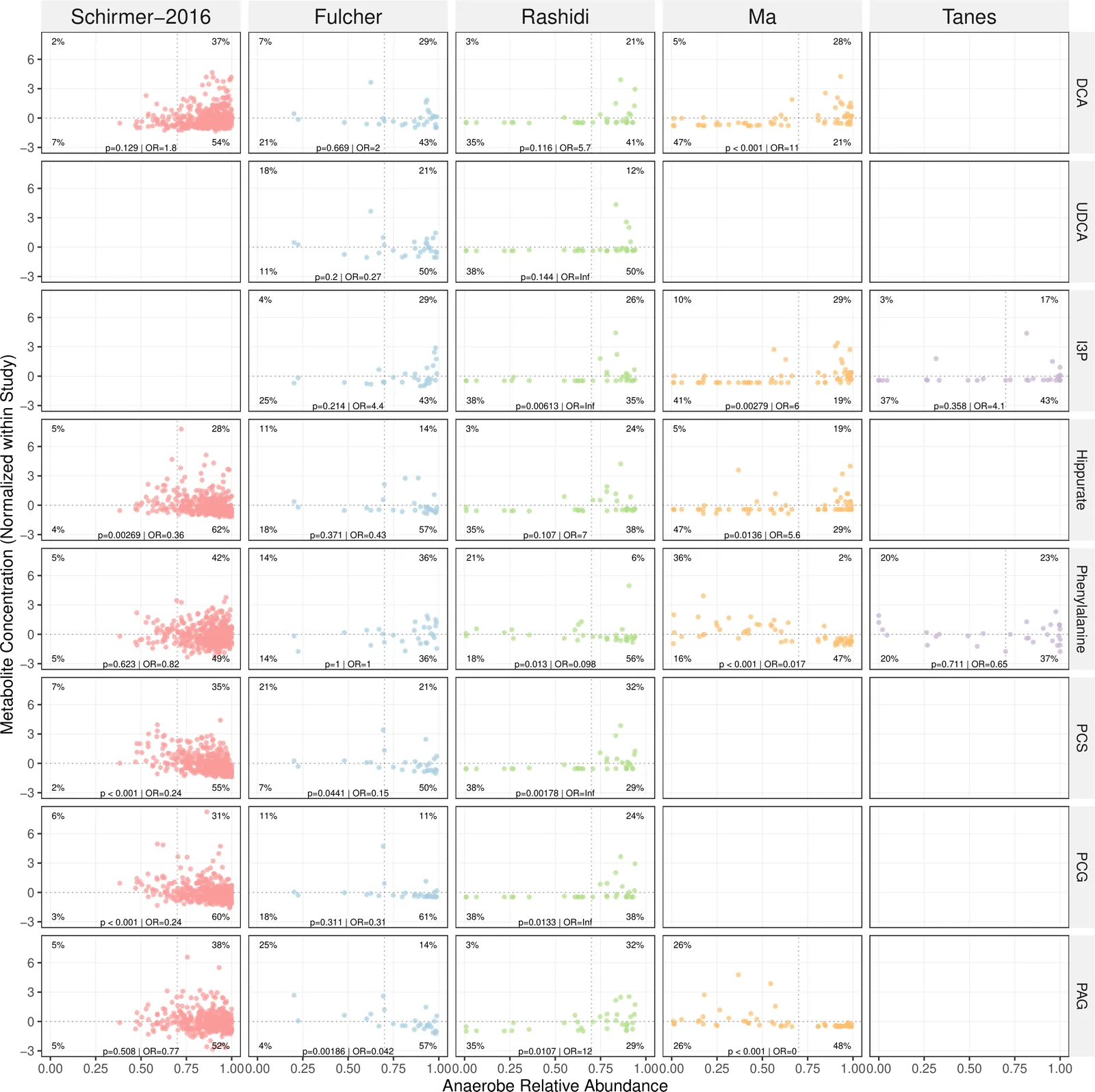
Normalized metabolite concentrations and anaerobe RA. Relationships between metabolite levels correlated with anaerobe RA (included studies had minimum Spearman >0.40 in at least one metabolite). Quadrants are split based on median metabolite level (in each study, *y*-axis) and anaerobe RA of 0.70 (*x*-axis), with percentage of samples shown in each quadrant. *P*-values are from Fisher’s exact tests. DCA, deoxycholate; UDCA, ursodeoxycholate; I3*P*, indole-3-propionate; PCS, p-cresol sulfate; PCG, p-cresol glucuronide; PAG, phenylacetylglutamine

Statistical analysis was performed in R v4.3.1. Metabolite associations were calculated using Spearman correlations. For conventional meta-analysis, point estimates were calculated as the ratio of the median in the high anaerobe RA group over the median in the low anaerobe RA group. Similar strategies have been utilized elsewhere, with some modeling data showing that median strategies may be preferable to transformation strategies (32–34), when outcome data are skewed (33). Confidence intervals were calculated using a non-parametric bootstrap (1,000 replicates). For pooled estimates of individual metabolites, an inverse-variance weighted fixed-effects model was used. Ratios were visualized on the log scale, but estimates are reported untransformed. Details are shown in the results section and in the legends of Fig. 2 as well as Fig. S4. To visualize the relationship of metabolites, samples were z-score-normalized. L-shaped associations were evaluated using Fisher’s exact test to estimate the odds ratios. Details are shown in the legend of Fig. 3.

**Fig 4:**
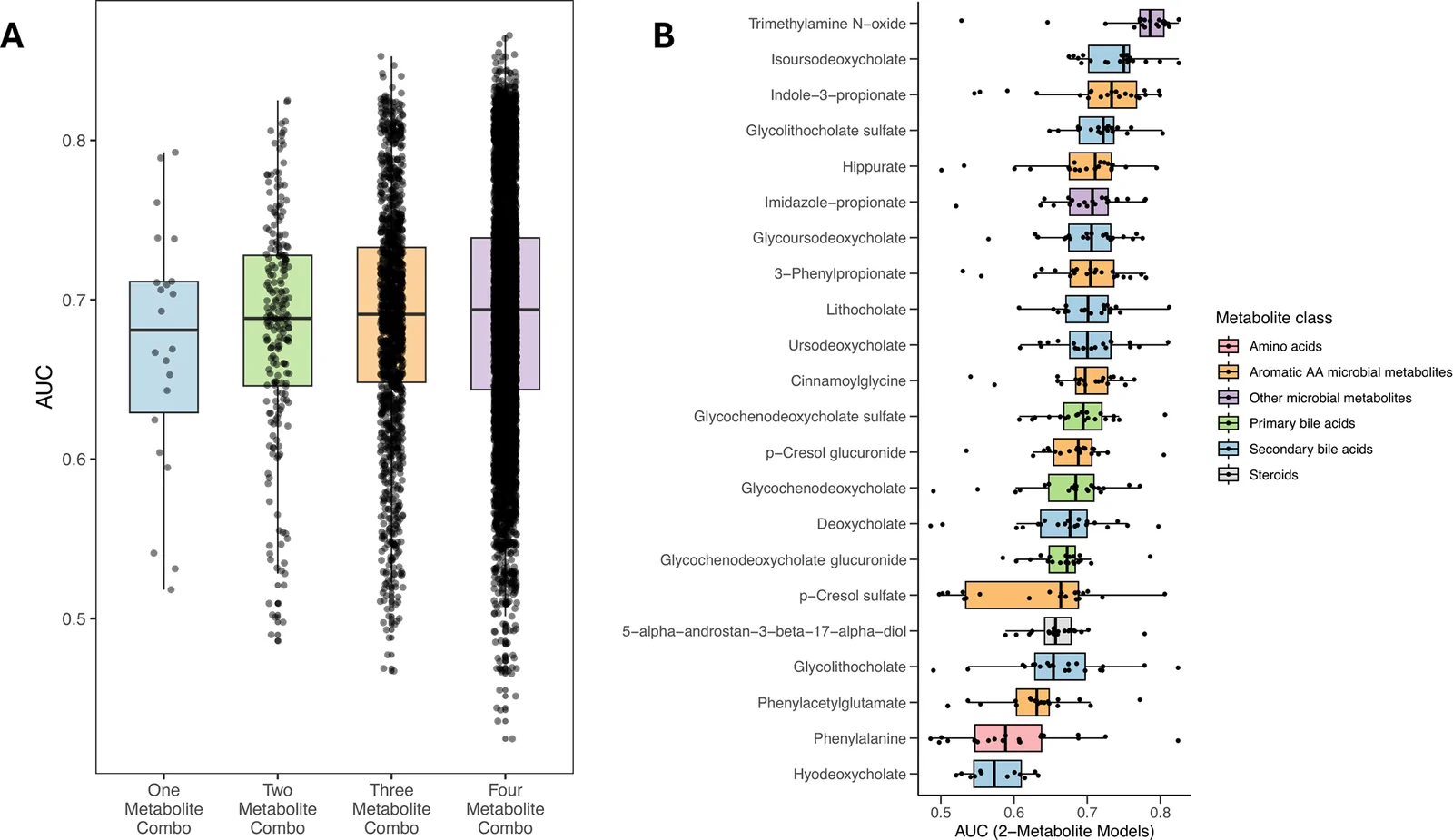
Prediction of low anaerobe RA using microbial metabolites. (**A**) Areas under ROC curves for individual metabolites and all metabolite combinations to predict anaerobe RA <0.70. (**B**) Boxplots showing the distribution of AUCs for all two-metabolite combinations involving the reference metabolite. Metabolites are ordered by median AUC. Metabolites are colored by metabolite class. AA, amino acid; AUC, area under the curve.

For patient-level meta-analysis, the results were normalized based on the reference metabolite, histidine. The performance of receiver operating characteristic (ROC) curves was quantified by AUC and McFadden pseudo-*R*^2^. To account for increasing complexity of models, we computed adjusted pseudo-*R*^2^ and Bayesian information criterion (BIC). Combinations were ranked primarily by BIC, penalizing larger, more complex models. Predictions were assessed on out-of-bag samples from 500 bootstrap resamples to compute 95% confidence intervals for sensitivity and specificity at each threshold. Details can be found in Results and in the legend for Fig. 4.

**Fig 5:**
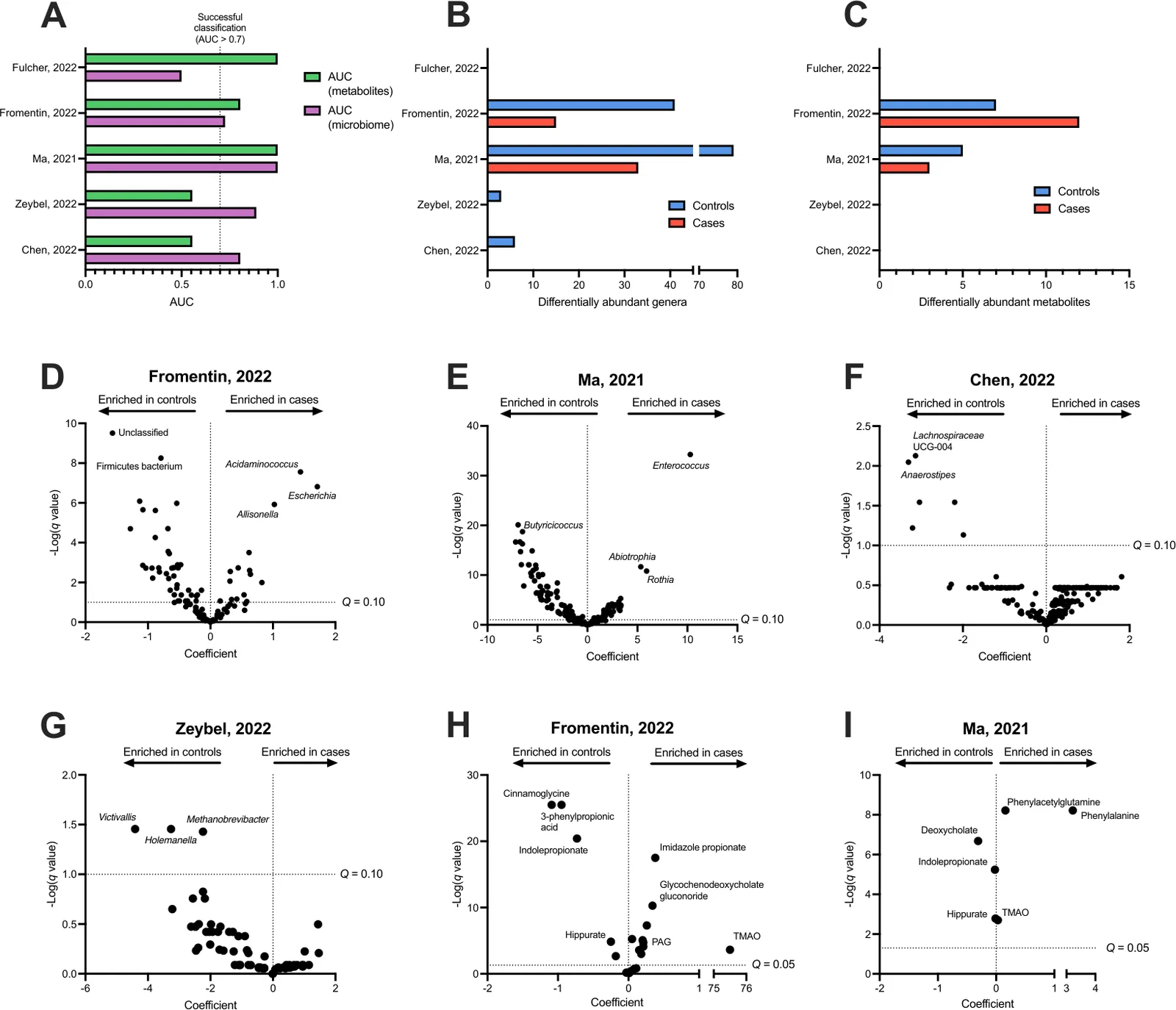
Differentiation of disease cases from controls using the gut microbiome and peripheral microbial metabolites. (**A**) AUCs of gut microbiome and metabolite random forest classifiers for cases and controls, with the threshold for successful classification at AUC = 0.7. The number of differentially abundant (**B**) bacterial genera and (**C**) microbial metabolites in cases and controls. (**D–G**) Association between individual bacterial genera and case-control status as determined using MaAsLin2, with an FDR-corrected *q* value of 0.10 used as the threshold for significance testing. (**H and I**) Association between individual candidate metabolites and case-control status as determined using Mann-Whitney *U* tests, with an FDR-corrected *q* value of 0.05 used as the threshold for significance testing. TMAO, trimethylamine-N-oxide; PAG, phenylacetylglutamine.

For case-control analysis, the performance of microbiome and metabolite random forest models was compared with Delong’s test. Differentially abundant bacterial genera were measured between cases and controls using Multivariable Association with Linear Models 2 (MaAsLin2), with case-control status as a fixed effect and a false discovery rate (FDR)-adjusted *q* value threshold of 0.10 for significance testing. Metabolite abundances were compared with Mann-Whitney *U* tests, and *P*-values were adjusted for multiple comparisons with the FDR. For metabolite comparisons, an FDR-adjusted *q* value threshold of 0.05 was used for significance testing. Details can be found in Results, as well as in the legends for Fig. 5 and Fig. S7.

**Fig 6:**
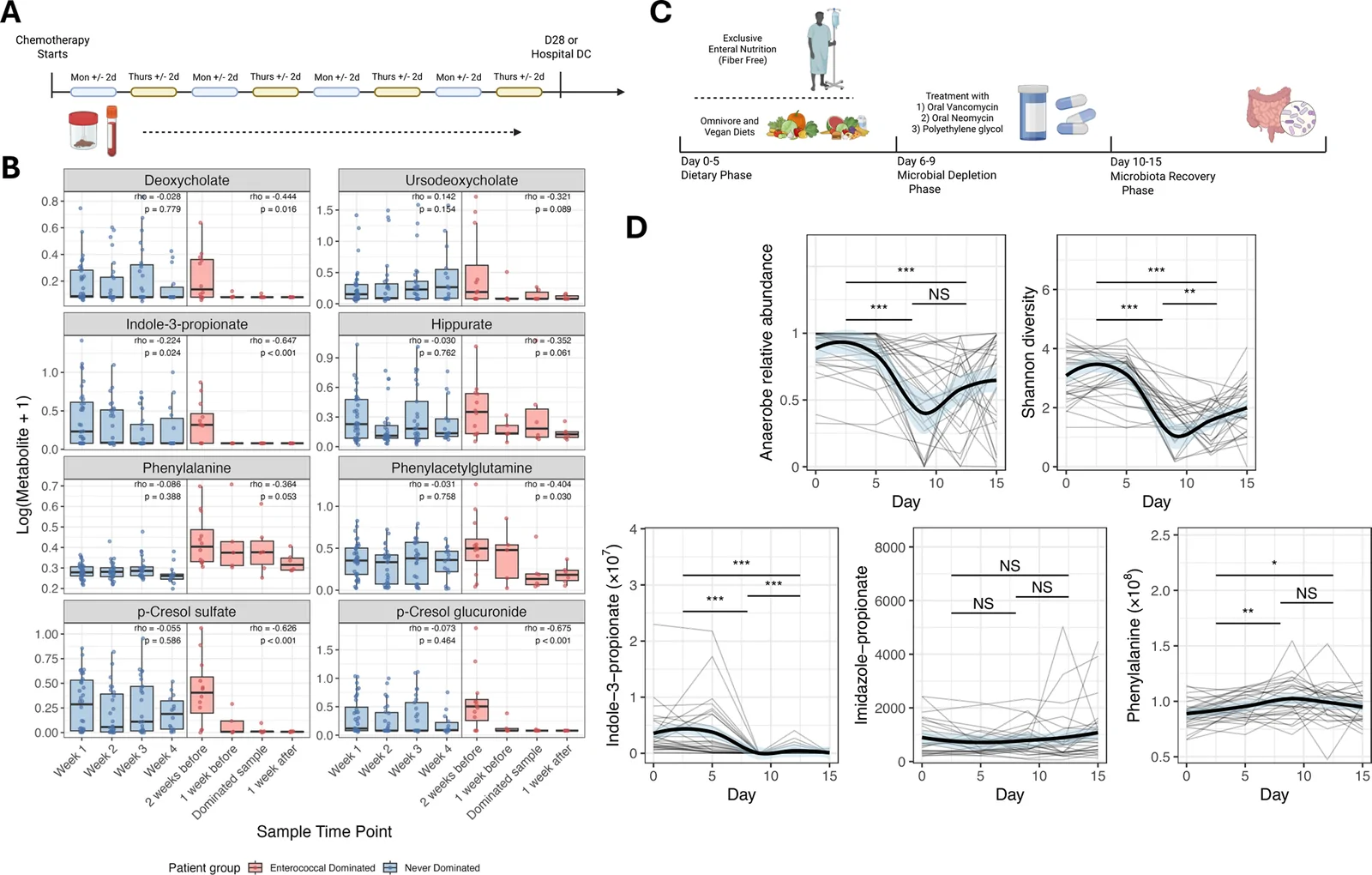
Longitudinal microbial metabolite dynamics. (**A**) Rashidi et al. study design: patients with acute myeloid leukemia (AML) were enrolled at the start of induction chemotherapy, with blood and stool collected twice weekly until hospital discharge or day 28. (**B**) Metabolite abundances stratified by *Enterococcus* domination status in the study by Rashidi et al. Boxplots show log₁₀(metabolite + 1) across time bins, aligned to the first dominated sample for dominated patients; points denote individual samples. Spearman ρ and *P*-values summarize within-group temporal trends. The eight metabolites shown had |ρ| >0.4 with anaerobe relative abundance in at least one study. (**C**) Tanes et al. study design: Participants were randomized to a dietary intervention (days 0–5), then all were given antibiotics (days 6–8), followed by gut microbiome sampling (days 10–15). (**D**) Longitudinal trajectories of ecological metrics and metabolite levels are shown. Thin lines show individual subject-level measurements, with thick lines showing locally estimated scatterplot smoothing and 95% CIs. Pairwise differences between bins (0–5, 6–9, and 10–15) were tested by Wilcoxon rank-sum (*P* > 0.05, not significant [NS]; *P*  <  0.05, *; *P* < 0.01, **; *P* < 0.001, ***).

For longitudinal analysis in a study by Rashidi et al., linear mixed-effects models evaluated temporal trajectories, including fixed effects for patient group, study day, and their interaction, and a random intercept per patient. The interaction term tested for group differences in longitudinal trends. For Tanes et al., pairwise comparisons between consecutive bins were conducted using Wilcoxon rank-sum tests, with significance annotated on plots. Details can be found in Results and in the legends for Fig. 6 as well as Fig. S8 and S9. Unless otherwise stated, statistical significance was defined as two-sided α <0.05.

### Software

FASTQ files were processed with Quantitative Insights Into Microbial Ecology 2 (QIIME 2, v2024.2) using default parameters (35). Quality filtering and denoising of reads were performed using DADA2 (36). ASVs were taxonomically annotated using the SILVA 132 99% database (24). Adapters were trimmed using Trimmomatic version 0.39 (37), and human reads were removed using KneadData version 0.12.0. Taxonomic annotation was performed using Metagenomic Phylogenetic Analysis (MetaPhlAn) version 4.1.1 (38).

Analyses were performed in R v4.3.1 using dplyr 1.1.4 (39), ggplot2 3.5.1 (40), pROC 1.18.5 (41), patchwork 1.3.1 (42), ggnewscale 0.5.2 (43), purrr 1.0.2 (44), caret 7.0-1 (45), randomForest 4.17-1.2 (46), nlme 3.1-166, (47), ecodist 2.1.3 (48), vegan 2.6-10 (49), FactoMineR 2.11 (50), missMDA 1.19 (51), and MaAsLin2 1.16.0 (52).

## RESULTS

### Systematic review

In total, 6,054 unique abstracts were screened; 5,191 studies were deemed irrelevant, and 859 studies underwent full-text review. The full search strategy is shown in Table S1; 150 studies presented only direct comparisons between metabolites and microbiome composition, of which 33 studies included at least 250 participants for a total of 32,607 participants. The most common populations studied were individuals with cardiovascular or metabolic diseases (12 studies, 13,639 participants). Healthy cohorts were next most common (10 studies, 9,402 participants). Other populations included individuals with infection, rheumatologic, endocrine, renal, neurologic, and liver diseases. Study details are available in Supplemental material (“Box A Studies”). Using these studies, we identified candidate microbial metabolites or host-microbe co-metabolites by assessing reported associations between circulating metabolites and microbiome features. Our goal was to reduce the number of candidate metabolites and enrich for those most likely to be generalizable to diverse populations. Metabolites that had associations with groups of bacteria of interest (e.g., obligate anaerobes and potential pathogens) or with global microbiome compositional indices (e.g., alpha-diversity) in multiple studies scored more points and were more likely to be included (see Supplemental methods for details of the selection process). This yielded 22 candidate metabolites that were selected based on their correlations with microbiome composition (box A; Fig. 1). The selection scoring tool is shown in Table S2, and classification of candidate metabolites is in Table S3. Candidate metabolites derived from this analysis were included in a meta-analysis of studies reporting both metabolite concentrations in plasma or serum and stool microbiome composition.

Microbiome and metabolomic data were available at an individual patient level for 57 studies; 33 of these studies required primary metabolomic data extraction and were excluded. The remaining 24 studies were reviewed for inclusion in the primary analysis. Of the 24 studies reviewed, five further studies were excluded: three did not provide metadata to match metabolite and microbiome data (53–55), one did not provide microbiome data in the referenced database (56), and one provided microbiome data that, upon analysis, was markedly different than the published results and not consistent with a human gut microbiome (57). The authors of these studies were contacted, and only one responded (54), indicating that an external application was required to access matching patient-level details, and therefore, the study was excluded. Nineteen eligible studies (30, 31, 58–74) were included in the primary analysis (box B in File S3), with 3,242 participants and 2,999 complete cases (values present for both metabolites and microbiome at the same sampling time point). Three studies did not report any candidate metabolites from our metabolite selection strategy (box A in Fig. 1) and were not included in subsequent analyses.

Disrupted microbiome cases were defined as those with an anaerobe RA below 0.70, which approximates the lowest 10% of the distribution of anaerobe RA for healthy individuals based on published data (Supplemental methods). This cutoff approximated the 10th percentile of healthy controls within the individuals included in this systematic review (0.72; Fig. S1). Six studies included fewer than five cases with low anaerobe RA and were therefore excluded. In total, 10 studies, representing 2,270 participants and 1,903 complete cases, were included in the study-level meta-analysis, while 16 studies, representing 2,622 patients (2,194 complete cases), were included in the patient-level meta-analysis. Study details by analysis are shown in Table S4.

### Study characteristics

Characteristics of the included studies are shown in Table 1. Healthy populations and a variety of disease states were captured, with cardiovascular disease and cancer being the most common (both 3/19, 15.8%). Only two studies (Rashidi et al*.* [30] and Tanes et al*.* [31]) reported participant-level antibiotic use before or during the study period. The median number of peripheral metabolites reported across studies was 655 (range 5–10,026). Candidate metabolites reported in each study are shown in Tables S4 and S5. Anaerobe RA and Shannon diversity index (SDI) varied across studies (Fig. S2A and B). The median SDI ranged from 1.65 to 5.18. Anaerobe RA varied, with medians between 0.64 and 0.99 (Fig. 2A). Correlation between SDI and anaerobe RA is shown in Fig. S2C. Two studies (Rashidi et al*.* [30] and Ma et al*.* [74]) had median anaerobe RA <0.80. Twelve studies had at least five cases with anaerobe RA <0.70.

**TABLE 1:** Overview of the included studies^*a*^

#	Author	*N* (complete)	Age (IQR)	Sex (% male)	Abx reported	Disruption features in >5 pts	Country/region	Population	Cases	Comparison/controls
1	Wang et al. (73)	40 (35)	36 (30–43)	79.5%	No	No	China	Liver	Hepatitis B	Healthy controls
2	Bishehsari et al. (72)	123 (118)	44 (34–53)	27.6%	No	No	USA	CV	MetS	–
3	Zeybel et al. (71)	41 (41)	40 (33–45)	68.3%	No	No	Turkey	Liver	NAFLD w steatosis	NAFLD without steatosis
5	Lin et al. (70)	517 (498)	53 (51–54.5)	0%	No	Anaerobe RA <0.7	China	Hormonal	Menopause	–
6	Schirmer et al. (69)	488 (441)	23 (21–27)	43.2%	No	Anaerobe RA <0.7	Western Europe	Healthy	Healthy	–
7	Chen et al. (68)	45 (45)	–	–	No	Anaerobe RA <0.7	China	Oncology	Lung cancer	Healthy controls
8	Fulcher et al. (67)	28 (28)	–	100%	No	Anaerobe RA <0.7	USA	Infection	HIV acquisition	Longitudinal
9	Fromentin et al. (66)	1,087 (992)	60 (52–66)	61.4%	No	Anaerobe RA <0.7 Dominance >30%	Western Europe	CV	CV diseases	Between CV diseases
10	Tanes et al. (31)	30 (30)	25.5 (24–37)	–	Yes	Anaerobe RA <0.7 Dominance >30%	USA	Healthy	Diets	Longitudinal interventional design
11	Sereti et al. (65)	137 (130)	62 (55–68)	97.8%	No	No	The Netherlands	Infection	PWH	Healthy controls
12	Ma et al. (74)	59 (58)	–	–	No	Anaerobe RA <0.7 Dominance >30%	China	Renal	Renal transplant	Healthy controls
13	Uema et al. (64)	140 (140)	60 (47–67)	50%	No	Anaerobe RA <0.7	China	CV	MetS	Healthy controls
15	Riwes et al. (63)	25 (25)	–	–	No	No	USA	Oncology	ASCT	Longitudinal of various diets
16	Gershuni et al. (62)	48 (44)	30 (26.5–34.5)	0%	No	No	USA	Healthy	Obesity in pregnancy	Normal BMI in pregnancy
18	Shoer et al. (61)	152 (148)	–	–	No	Anaerobe RA <0.7	Israel	Healthy	Diet	Long. of various diets
19	Rashidi et al. (30)	46 (34)	59 (50–68.5)	64.9%	Yes	Anaerobe RA <0.7 Dominance >30%	USA	Oncology	AML	Long. through AML induction course
20	Schirmer et al. (60)	241 (85)	13 (10–15)	55.6%	No	Anaerobe RA <0.7 Dominance >30%	USA	Gut	IBD	Weeks from UC diagnosis
21	Levi et al. (59)	94 (68)	37 (30–46.5)	31.9%	No	Anaerobe RA <0.7	Israel	Neuro	MS	Healthy controls
22	Kang et al. (58)	38 (38)	10.9 (8.8–12.7)	86.8%	No	No	USA	Neuro	Autism	Healthy controls

^*a*^ Age is median in years (IQR); a cell is recorded as NA if age or sex is not reported at the patient level for that study. *N* is the total number of unique patients. Complete cases are unique samples with paired metabolite and gut microbiome data. Dominance is defined as the presence of a non-obligate anaerobe with RA >30% and at least twice the next most prevalent taxon. IQR, interquartile range; CV, cardiovascular; MetS, metabolic syndrome; NAFLD, non-alcoholic fatty liver disease; RA, anaerobe relative abundance; Abx, antibiotics; PWH, persons with HIV; ARV, antiretrovirals; ASCT, allogeneic stem cell transplant; AML, acute myeloid leukemia. – indicates that the data were not reported.

### Metabolite associations and meta-analysis

We hypothesized that alterations in peripheral metabolites would be most pronounced in individuals with highly disrupted microbiomes and reasoned that associations between anaerobe relative abundance and metabolite levels would be strongest in studies that included individuals with both preserved and depleted gut anaerobes (i.e., studies with greater variance in anaerobe RA). Anaerobe RA for each study is presented in Fig. 2A and is ordered by increasing variance. Values for anaerobe RA variance are shown in Table S7. Correlations between each metabolite and anaerobe RA are shown in Fig. 2B and C. Studies with low variance in anaerobe RA had weak correlations between metabolites and anaerobe RA, and this tended to increase as anaerobe RA variance increased. Five studies, Schirmer et al. (69), Fulcher et al. (67), Rashidi et al. (30), Ma et al. (74), and Tanes et al. (31), had at least one metabolite with a moderate Spearman correlation (>|0.4|) with anaerobe RA, which included DCA, UDCA, I3P, hippurate, phenylalanine, PCS, PCG, and PAG. The strongest negative and positive correlations with anaerobe RA were both from Ma et al*.* (74), in which PAG was negatively correlated (Spearman = −0.65), and I3P was positively correlated (Spearman = 0.58).

Methods for metabolite quantification and normalization differed across studies. To control for the variable scales of each metabolite, we calculated the median ratio between metabolites in the low and high anaerobe RA groups (Fig. 2D), a strategy that has been used previously (32–34). We again noted that larger ratios were generally seen in studies with more variance in anaerobe RA. Metabolites that were products of obligate anaerobe metabolism generally had larger ratios than other metabolites. The predicted direction of metabolite associations with anaerobe RA is shown in Table S6. We performed sensitivity analyses using anaerobe RA cutoffs below the bottom 5% of healthy individuals (cutoff = 0.6) and above the bottom 15% (cutoff = 0.8) and observed similar associations (Fig. S3).

In study-level meta-analyses, log median ratios of two candidate metabolites were significantly different between high and low anaerobe RA participants: DCA (pooled estimate = 1.62-fold, 95% CI: 1.32–2.04) and PCG (pooled estimate of 0.78-fold [95% CI: 0.68-fold to 0.89-fold], Fig. S4). Other candidate metabolite ratios did not significantly differ between high- and low-anaerobe RA participants.

In studies with a greater proportion of patients with anaerobe-depleted microbiomes, products of anaerobic microbial metabolism (I3P, DCA, UDCA, and hippurate) were more strongly associated with higher anaerobe RA (Fig. 3). Given that relationships between metabolites and anaerobe RAs were non-linear (i.e., decreased markedly at lower anaerobe RAs), we evaluated associations using an anaerobe RA threshold of 0.70. Across cohorts, associations between mean metabolite values and this threshold were strongest for DCA (odds ratio [OR] ranged from 1.8 [95% CI 0.86–4.1, *P* = 0.13] to 11.4 [95% CI 2.6–72.7, *P* < 0.001]), and I3P (OR ranged from 4.1 [95% CI 0.37–218.0, *P* = 0.36] to infinite [95% CI 1.63–infinite, *P* = 0.006]). Conversely, PCS, PCG, and PAG generally showed inverse associations with anaerobe RA, with the exception of Rashidi et al. (30). These effects were most notable for PAG (OR ranged from 0.77 [95% CI 0.38–1.55, *P* = 0.51] to 0 [95% CI 0–0.18, *P* < 0.001]). Effect sizes generally increased with higher variance in anaerobe RA across cohorts.

### Patient-level meta-analysis

We next generated logistic regression models of metabolite levels to predict low anaerobe RA (<0.70). To control for differences in scaling between studies and unitless reporting of metabolites, all metabolite levels were normalized by histidine, which was reported in all studies and had the lowest average coefficient of variance across studies (see Supplemental methods for further details). Models included individual metabolites and combinations of up to four different metabolites. When penalizing for model complexity using BIC, two-metabolite combinations provided the best balance between predictive model performance and parsimony (Fig. 4A). AUC curves for predicting low anaerobe RA using individual peripheral metabolites are shown in Fig. S5. The best-performing individual metabolites were isoursodeoxycholate (IUDCA; AUC 0.79, 95% CI 0.72–0.85) and trimethylamine-N-oxide (TMAO; AUC 0.79, 95% CI 0.75–0.83). Among all two-variable models, models that included TMAO had the best median performance (median AUC 0.79, interquartile range [IQR] 0.77–0.81; Fig. 4B), and inclusion of a secondary bile acid in two-variable models was associated with improved performance (Fig. 4B). Combinations such as phenylalanine + glycolithocholate (GLCA) and IUDCA + TMAO produced the strongest predictions (Fig. S6A and B) among two-metabolite combinations. Sensitivity analysis using alanine-normalized metabolite levels produced similar results (examples are provided in Fig. S6C and D). Sensitivity analyses were also performed for anaerobe RA cutoffs of 0.65 and 0.75 (approximations of the bottom 5% and 15% of healthy microbiomes), as well as more extreme cutoffs of 0.5 and 0.8 (Fig. S6E through H).

### Application of peripheral metabolite levels to diagnostic scenarios

#### Case-control prediction

To test whether circulating metabolites and gut microbiome composition could distinguish between health and disease, we selected all studies with data for disease cases and controls that reported at least two candidate microbial metabolites. Six studies across diverse participant populations were eligible for inclusion in this analysis (Table S5). We used random forest classification models with genus-level gut microbiome data or peripheral microbial metabolites to classify cases and controls in each study. Only participants with paired metabolite and microbiome data were included to allow direct comparison of model performance. One participant from Ma et al. who did not have metabolite data was excluded. Participant IDs could not be matched between metabolite and microbiome data from Kang et al., and hence, this study was excluded. Case and control status was successfully predicted (AUC > 0.70) in 4/6 microbiome classifiers and 4/6 metabolite classifiers (Fig. 5). Classification of cases and controls in testing data sets is presented in Table S7. Clustering of cases and controls based on microbiome or metabolite data followed similar patterns as the random forest classifiers; studies with successfully classified cases and controls tended to have clearer separation of cases and controls (Fig. S7). We then used MaAsLin2 (for microbiome data) or Mann-Whitney *U* tests (for metabolomic data) with FDR correction to test for differentially abundant features between cases and controls. Gut commensals were more abundant in controls than in cases, and cases were enriched with potentially pathogenic taxa including *Enterococcus* and *Escherichia* (Fig. 5). Differentially abundant metabolites between cases and controls were observed in two studies (74, 66). Although these studies measured different metabolites, cases in both studies had elevated TMAO and PAG, and controls had elevated I3P and hippurate (Fig. 5). Peripheral metabolites performed similarly to the gut microbiome for distinguishing between cases in these data sets (all *P* > 0.05 by Delong’s test).

#### Analysis of longitudinal sampling with acquired anaerobe loss

We reasoned that one potential use for plasma-based assays predicting microbiome disruption would be to identify both risk-associated microbiomes (i.e., those associated with a specific outcome in a given population) and for the assessment of iatrogenic perturbation of microbiomes (e.g., after antibiotics or other interventions). Two studies (30, 31) collected samples longitudinally and included participants who developed microbiome disruption during sampling, one in which microbiome disruption has been associated with infection and death (acute myeloid leukemia [AML] patients in Rashidi et al.), and another in which antibiotics and osmotic laxatives were given to healthy volunteers (Tanes et al.). We assessed candidate metabolites in both populations during repeated sampling and compared the trajectories of analytes with and without acquired *Enterococcus* domination (a risk-associated ecological state (11, 12) and before, during, and after antimicrobial/laxative exposure (an iatrogenic microbiome disturbance) in these two studies. Our findings are shown in Fig. 6.

In the AML cohort, a large proportion of study participants were given antibiotics during their acute illness, which was associated with decreased microbiome diversity and anaerobe RA (Fig. S8). Plasma metabolite levels decreased significantly only in individuals with acquired *Enterococcus* dominance, and not in those without this acquired ecological disruption (Fig. 6B). Of interest, we observed significant reductions in metabolites that were negatively associated with anaerobe RA in the meta-analysis, such as PAG, PCS, and PCG. These metabolites (or precursors to these metabolites) can be produced by facultative anaerobes when substrates accumulate with obligate anaerobe loss, a process which may be disrupted by antibiotic-mediated pathogen depletion. Importantly, all participants in this study were given fluoroquinolone prophylaxis, and consequently, expansion of facultative anaerobic taxa was rare (Fig. S8), potentially explaining this observation. Individual patient metabolite dynamics are shown in Fig. S9.

We next examined the relationship between metabolites and microbiomes after a dietary intervention and antibiotic and laxative exposure in healthy volunteers in the study reported by Tanes et al*.* (31). Antibiotics (vancomycin 500 mg orally q6h and neomycin 1,000 mg orally q6h) and oral PEG were administered to participants on days 6, 7, and 8 (Fig. 6C). As expected, profound drops in diversity and anaerobe RA during and after administration of antibiotics and PEG were evident (Fig. 6D). Concordantly, the metabolite I3P decreased to undetectable levels in all participants, while, as expected, the same effect was not seen for imidazole propionate or phenylalanine (Fig. 6D).

These data indicate the potential utility of plasma metabolites for detecting longitudinal disruptions of microbiomes within individuals, including for the detection of acquired risk-associated or iatrogenic microbiome perturbation.

## DISCUSSION

In this systematic review and meta-analysis, we found that circulating microbial metabolites decreased in association with profound gut anaerobe loss, a finding driven by studies where a significant proportion of participants had highly disrupted microbiomes. This association was true across diverse disease states and populations. Plasma concentrations of products of metabolism by obligate anaerobes, including secondary bile acids (SBAs), indole derivatives, and hippurate, were most consistently associated with microbiome disruption. Products of facultative anaerobe metabolism generally increased with anaerobe loss and overgrowth of pathogenic taxa, but this may be context-dependent. Finally, substrates of anaerobic microbial metabolism had the weakest associations with microbiome composition.

Several studies have now attempted to use peripheral biomarkers to infer gut microbiome composition. These studies have identified associations between gut microbiome features, including alpha-diversity, and peripheral microbial metabolites such as SBAs, amino acid derivatives, 3-PPA, I3P, cinnamoylglycine, PCS, and hippurate (75–81). However, inter-study heterogeneity in patient populations and the methods used for metabolite quantification make it challenging to translate these findings into clinically actionable tools. In our meta-analysis, we also found that SBAs (i.e., DCA and UDCA) and indole metabolites (i.e*.*, I3P) were reduced with anaerobe depletion in diverse populations. Primary bile acids (PBAs) are deconjugated by taxonomically diverse organisms and then converted to SBAs (82, 83), a process largely restricted to obligate anaerobes within the *Clostridia* class (84, 85). I3P is formed in the gut via reductive microbial metabolism of tryptophan, a process carried out by a restricted group of obligate anaerobes (86). Furthermore, we show that alterations in peripheral microbial metabolites are largely restricted to individuals with profound depletion in gut anaerobes, suggesting that peripheral metabolite screening to infer microbiome disruption may be most useful in clinical populations at risk of major gut microbiome depletion.

Products of metabolism by facultative anaerobes tended to increase with anaerobe loss, but not uniformly across studies. For example, phenylacetate (PAA), the precursor to PAG, is produced primarily by obligate anaerobes, such as *Clostridia* and *Bacteroides* species, before being conjugated in the liver. When obligate anaerobes are depleted, phenylalanine is rerouted to PAA via facultative anaerobes (87). Notably, in one included study (Rashidi et al. [30]), PAG decreased with gut microbiome disruption, rather than increased. Of note, the patients within this study were all treated with fluoroquinolone antibiotics and consequently had very low RA of *Enterobacteriaceae*. The variability in this association may represent biologically informative heterogeneity, but this needs to be assessed in larger and specifically designed studies. TMAO, which can be produced via facultative anaerobe metabolism, was also among the strongest predictors of low anaerobe RA and was enriched in disease cases vs controls, indicating its potential utility to identify disease-associated disrupted microbiomes.

Our study has several important limitations. First, only a minority of studies included populations with a large proportion of individuals with microbiome disruption. Second, our initial candidate-selection strategy was designed to enrich for representation in the maximum number of studies, potentially excluding informative metabolites represented in fewer published reports. Third, metabolites were largely reported as batch-normalized, unitless values, preventing a direct analysis of merged data. We elected not to normalize values by study (e.g., through *z*-score normalization) to avoid obscuring between-study drivers of metabolite-microbiome associations, but instead normalized by histidine values. While histidine may vary with disease states, sometimes showing a decrease in metabolic diseases (88), these variations are generally modest in comparison to the variations seen in microbial metabolites (89). Our analysis normalizing by alanine demonstrated similar findings, where alanine is expected to increase in metabolic diseases (90). Fourth, we did not uniformly reprocess metabolomic data and instead relied on metabolite methods and annotations provided by the primary studies. This likely introduced heterogeneity, which would bias against finding associations but potentially increase the generalizability of findings. Fifth, we used simplified indicators of microbiome disruption (anaerobe relative abundance), with cutoffs based on data distributions/prevalence rather than a specific biological association. There may be specific and preserved relationships between metabolites and individual families or taxa not detected with such a broad ecological indicator. Finally, the populations included in our analysis are heterogeneous and include geographically distinct cohorts with different health statuses, variable diets, and exposures. Again, this would be expected to decrease the strength of associations compared to more homogeneous cohorts. Importantly, numerous exposures, including diet, can affect the plasma metabolome as well as the microbiome. Notably, in one included study (Tanes et al.), the effect of three dietary interventions can be directly compared to the effect of antibiotics and laxatives. The observed impact of the antibiotic/laxative combination greatly exceeded the between-diet-group differences in the assayed metabolites, suggesting that the overall associations may be robust to at least some dietary interventions.

### Conclusions

Circulating microbial metabolites were consistently associated with gut anaerobe loss and ecological collapse, provided approximately equivalent discrimination between cases and controls as disrupted microbiomes in a series of health states, and provided informative indicators of within-individual treatment-induced gut microbiome disruption. While not intended to replace direct gut microbiome assessments using sequencing or culture, our analysis establishes peripheral microbial metabolites as potentially informative biomarkers of gut microbiome disruption. Targeted validation in well-designed observational and interventional cohorts is now required and would likely be most informative in populations in which a significant proportion have baseline or acquired highly disrupted microbiomes, particularly those linked to health outcomes or treatment responses, such as those with critical illness, exposure to broad-spectrum antibiotics, and hematological malignancy.

## Data Availability

Compiled microbiome and metabolite data from each study are available as supplemental material. The original and full data with accession numbers for extraction from each study are available from the original study and as referenced in the supplemental table. Code generated during this study is available at https://github.com/taylorkain1/metabolome-SR. This study did not generate any new materials or unique reagents.

## SUPPLEMENTAL MATERIAL

Supplemental material mbio.01927-26-s0001.docx Supplemental methods, figures, and tables.

File S1 mbio.01927-26-s0002.xlsx Summary of all studies from box B (used to do the primary analysis).

File S2 mbio.01927-26-s0003.xlsx Summary of all studies from box A (used to generate a list of candidate metabolites).

File S3 mbio.01927-26-s0004.xlsx Details of the methods used for metabolomics analysis of each study, as well as the naming conventions and certainty (where available) for each metabolite in each study.

ASM does not own the copyrights to Supplemental Material that may be linked to, or accessed through, an article. The authors have granted ASM a non-exclusive, world-wide license to publish the Supplemental Material files. Please contact the corresponding author directly for reuse.
