# Supplementary material for: Peripheral microbial metabolites as indicators of gut microbiome disruption: systematic review and meta-analysis
Source: mBio. 2026 Oct 7;17(11):e01927-26. doi: 10.1128/mbio.01927-26 (PMC13753740; doi:10.1128/mbio.01927-26)
Supplement: Supplementary file 1 [file mbio.01927-26-s0001.docx]

**Supplementary Methods**

One-hundred and fifty studies met inclusion criteria but did not have publicly available metabolite or gut microbiome data and instead made direct comparisons between metabolite and gut microbiome features. Candidate metabolites were selected from Box A of PRISMA (Figure 1). Of these, studies with less than 250 participants were excluded to decrease risk of low-level associations in non-generalizable populations. The remaining 33 studies were screened for associations between peripheral metabolites and gut microbiome features to generate a list of relevant metabolites which informed our primary analysis (Box A). These studies used a variety of methods to compare metabolite levels to gut microbiome composition, including spearman correlation, multivariable regression, and Least Absolute Shrinkage and Selector Operator (LASSO) regression. Gut microbiome features that were associated with metabolite levels included alpha diversity metrics (*e.g.*, Shannon diversity, richness) and the relative abundance of individual microbes or groups of microbes at varying taxonomic levels. “High yield” candidate metabolites were included in our meta-analysis if they appeared in at least two papers and were moderately correlated (*i.e.,* an absolute Spearman correlation coefficient of > 0.4) with commensal obligate anaerobes or potential pathogens (*e.g.*, *Enterobacteriaceae*). Metabolites were also included if they had a single moderate correlation and at least two other weak correlations (*i.e.,* an absolute Spearman correlation coefficient of > 0.2) or had a weak correlation with a global gut microbiome composition (such as diversity or richness) and at least a moderate correlation with a specific microbe in another study (defined as regression analyses with a p<0.05). Details of scoring for each metabolite are shown in supplementary Table S2.

The cut off for anaerobe relative abundance (RA) of 0.7 matched the healthy populations within the papers of our study, as seen in supplemental Figure S1. Including only studies of healthy participants, or only healthy controls from other studies, the bottom 10% of individuals had an anaerobe RA of 0.72 (rounded to 0.7). The 5% cut-off was 0.64 and the 15% cut-off was 0.76. The bottom 10% was selected to represent a sufficiently disrupted microbiome, but sensitivity analyses were performed at other anaerobe RA cut-offs as well, supplementary Figure S3 and S6. Studies with fewer than 5 samples meeting this criterion were excluded from conventional meta-analysis due to imbalance in groups.

Histidine was selected as the reference metabolite for normalization, since it: 1) was widely represented across studies, 2) had one of the lowest co-efficient of variations across all studies, and 3) is an essential amino acid and thus is less likely to be affected by gut microbiome composition or disease state. It was the metabolite with has the lowest average coefficient of variation across studies that was also included in all studies. The mean histidine level for each study was used to normalize the levels of individual metabolites for each sample. Sensitivity analyses were performed using alanine (a non-essential amino acid), as it had similar characteristics to histidine (supplementary Figure S5C and S5D).

The following antibiotics were classified as anti-anaerobic: amoxicillin-clavulanate, ampicillin-sulbactam, clindamycin, ertapenem, imipenem, meropenem, metronidazole, piperacillin-tazobactam, tigecycline, and oral vancomycin.

**Supplementary Figures**

**Figure S1 – Anaerobe relative abundance (RA) distribution across studies with only healthy cohorts of control patients included.** The thick red line represents the bottom 10% of patients across all studies (cut off 0.72), while the dotted red lines represent the bottom 5% (0.64) and bottom 15% (0.76) cut-offs.

**B**

**A**

**E**

**D**

**C**

**Figure S2 – Microbiome characteristics of studies.** A) Anaerobe relative abundance (RA) and B) Shannon diversity index (SDI) across all studies, ordered by median anaerobe RA. C) Correlation of anaerobe RA and SDI. D) Distribution of anaerobe RA between low (<0.70) and high anaerobe RA. E) Three studies with variation from the expected pattern were examined; estimates from the original manuscript matched calculated values.

**Figure S3 –** Sensitivity analyses at different anaerobe relative abundance (RA) cut-offs. Forest plots showing the median ratio between different anaerobe RA cut-offs most positively and negatively correlated metabolites in each study. Confidence intervals were calculated using bootstrapping. A) cut-off = 0.6, B) cut-off = 0.8. *Indole-3-propionate in Ma *et al.* had an infinite median ratio as all patients in the low anaerobe RA group had a level of 0.

**Figure S4 –** Conventional meta-analysis for deoxycholate (left) and p-cresol glucuronide (right) showing the log (median ratios) between the high and the low anaerobe relative abundance groups with 95% confidence intervals calculated using bootstrapping with 1000 replicates. Inverse variance weighting used to calculate pooled estimate.

**Figure S5 – Individual metabolite ROC curves.** Individual candidate metabolite curves predicting low anaerobe relative abundance (RA) (<0.70). Models were fit using weighted logistic regression and evaluated by out-of-bag bootstrap resampling (500 iterations); shaded bands indicate 95% bootstrap confidence intervals.

AUC 0.83 (0.74-0.89)

AUC 0.83 (0.63-0.93)

AUC 0.82 (0.72-0.89)

AUC 0.83 (0.56-0.96)

AUC 0.84 (0.75-0.91)

AUC 0.82 (0.75-0.88)

AUC 0.85 (0.69-0.89)

AUC 0.83 (0.76-0.87)

**Figure S6 – Prediction of low anaerobe relative abundance (RA) using microbial metabolites after normalization using Alanine.** A-B) Receiver operating characteristic (ROC) curves for prediction of anaerobe RA (<0.70) using two metabolites after normalization for histidine. (A) Phenylalanine and glycolithocholate (GLCA); (B) Trimethylamine N-oxide (TMAO) and isoursodeoycholate (IUDCA). C-D) Sensitivity analysis after normalization for alanine. (C) Phenylalanine and GLCA; (D) TMAO + IUDCA. E-H) Sensitivity analyses for different anaerobe RA cut offs using TMAO + IUDCA as an example combination. E) cut-off = 0.65 (bottom 5% of healthy); F) cut-off = 0.75 (bottom 15% of healthy); G) cut-off = 0.5; H) cut-off = 0.8. Models were fit using weighted logistic regression with log-transformed metabolites and evaluated by out-of-bag bootstrap resampling (500 iterations); shaded bands indicate 95% bootstrap confidence intervals.

**Figure S7 –** Overall gut microbiome and circulating metabolome profiles based on case-control status. A-F) PCoA plots of genus-level gut microbiome profiles based on case-control status. G-L) PCA plots of candidate metabolite profiles.

**Figure S8 – Longitudinal gut microbiota dynamics in patients undergoing chemotherapy.** (A) Shannon diversity over time relative to start of chemotherapy, with points colored by anti-anaerobic antibiotic exposure (pre/no exposure: dark gray; during exposure: yellow-to-red gradient; post-exposure: blue-to-green gradient). LOESS/GAM smooth (black line) with 95% CI shown. (B) Mean relative abundance of obligate anaerobes, non-obligate anaerobes, and other or unclassified taxa across 5-day bins. Abx=antibiotics.

**Figure S9 –** Individual patient metabolite level dynamics over course of AML treatment in Rashidi *et al.* where thin red lines represent patients who had a sample dominated by *Enterococcus* at some point during the study, while black lines are patients who never became dominated. Thick lines represent locally estimated scatterplot smoothing (LOESS) to visualize the local relationship without assuming a global linear model. For each metabolite, longitudinal trends were assessed using linear mixed-effects models with subject-specific random intercepts, testing the interaction between time and domination status. P values and slopes correspond to the time × group interaction term. 3-PPA=3-phenylpropionate, DCA=deoxycholate, GCDCA=glycochenodeoxycholate, GLCA=glycolithocholate, GUDCA=glycoursodeoxycholate, I3P=indole-3-propionate, IUDCA=isoursodeoxycholate, LCA=lithocholate, PAG=phenylacetylglutamine, PCG=p-cresol glucuronide, PCS=p-cresol sulfate, TMAO=trimethylamine-N-oxide, UDCA=ursodeoxycholate.

**Supplementary Tables**

**Ovid MEDLINE: Epub Ahead of Print, In-Process & Other Non-Indexed Citations, Ovid MEDLINE® Daily and Ovid MEDLINE® <1946-Present>**

| 1 | exp Metabolomics/ | 28183 |  |  |  |
| --- | --- | --- | --- | --- | --- |
| 2 | exp Metabolome/ | 16268 |  |  |  |
| 3 | (micr* adj3 metab*).tw,kf. | 37221 |  |  |  |
| 4 | exp "Bile Acids and Salts"/ | 41072 |  |  |  |
| 5 | exp acetates/ or exp butyrates/ or propionates/ | 260348 |  |  |  |
| 6 | (short* chain adj2 fatty adj2 acid*).tw,kf. | 15751 |  |  |  |
| 7 | SCFA*.tw,kf. | 8294 |  |  |  |
| 8 | exp Benzoates/ | 178142 |  |  |  |
| 9 | Methylamines/ | 5366 |  |  |  |
| 10 | (trimethylamine adj2 oxide).tw,kf. | 3231 |  |  |  |
| 11 | TMAO*.tw,kf. | 2474 |  |  |  |
| 12 | exp tryptophan/ or exp tyrosine/ | 126311 |  |  |  |
| 13 | exp Fatty Acids, Omega-3/ | 29735 |  |  |  |
| 14 | exp fatty acids, omega-3/ or exp fatty acids, omega-6/ | 42690 |  |  |  |
| 15 | exp Caproates/ | 16598 |  |  |  |
| 16 | (pentadecanoic adj2 acid*).tw,kf. | 752 |  |  |  |
| 17 | heptanoic acids/ or heptanoates/ | 6168 |  |  |  |
| 18 | arginine/ or asparagine/ or citrulline/ or glutamine/ | 73214 |  |  |  |
| 19 | exp Dicarboxylic Acids/ | 84692 |  |  |  |
| 20 | (metab* adj2 phenotype*).tw,kf. | 7331 |  |  |  |
| 21 | Gastrointestinal Microbiome/ | 41136 |  |  |  |
| 22 | shannon diversity.tw,kf. | 1442 |  |  |  |
| 23 | (enterococ* adj3 domina*).tw,kf. | 153 |  |  |  |
| 24 | Proteobacteria/ | 3135 |  |  |  |
| 25 | (pathogen* adj3 domin*).tw,kf. | 1318 |  |  |  |
| 26 | (berger* adj2 parker*).tw,kf. | 83 |  |  |  |
| 27 | exp Microbiota/ and (gastro* or intestin* or colon* or gut* or stool*).tw,kf. | 48981 |  |  |  |
| 28 | 1 or 2 or 3 or 4 or 5 or 6 or 7 or 8 or 9 or 10 or 11 or 12 or 13 or 14 or 15 or 16 or 17 or 18 or 19 or 20 | 882741 |  |  |  |
| 29 | 21 or 22 or 23 or 24 or 25 or 26 or 27 | 56809 |  |  |  |
| 30 | 28 and 29 | 14066 |  |  |  |
| 31 | limit 30 to (humans and yr="2000 -Current") | 8487 |  |  |  |

**Table S1 –** Full OVID MEDLINE search strategy

| Metabolite | Number of Studies | Moderate Correlations | Weak Correlations |
| --- | --- | --- | --- |
| 3-Phenylpropionate (hydrocinnamate) (3-PPA) | 9 | 9 | 0 |
| 5-alpha-androstan-3beta,17alpha-diol disulfate | 2 | 2 | 0 |
| Cinnamoylglycine | 4 | 4 | 0 |
| Deoxycholic acid | 5 | 3 | 2 |
| Glycochenodeoxycholate | 4 | 3 | 1 |
| Glycochenodeoxycholate sulfate | 4 | 3 | 1 |
| Glycochenodeoxycholate glucuronide | 4 | 3 | 1 |
| Glycolithocholate | 2 | 2 | 0 |
| Glycolithocholate sulfate | 2 | 2 | 0 |
| Glycoursodeoxycholate | 8 | 6 | 2 |
| Hippurate | 4 | 3 | 1 |
| Hyodeoxycholic acid | 2 | 2 | 0 |
| Imidazole propionate | 3 | 3 | 0 |
| Indole-3-propionate | 5 | 4 | 1 |
| Isoursodeoxycholate | 4 | 4 | 0 |
| Lithocholate sulfate | 2 | 2 | 0 |
| p-Cresol glucuronide | 3 | 3 | 0 |
| p-Cresol sulfate | 7 | 7 | 0 |
| Phenylacetylglutamine (PAG) | 8 | 7 | 1 |
| Phenylalanine | 3 | 2 | 1 |
| Trimethylamine N-oxide (TMAO) | 6 | 1 | 3 |
| Ursodeoxycholate | 3 | 2 | 1 |

**Table S2** – Metabolite selection screening/scoring tool

List of other metabolites screened, but not meeting criteria for inclusion: 1-linoleoylglycerophosphocholine, 1-methylhistidine, 1-palmitoyl-2-gamma-linolenoyl-GPC (16:0/18:3n6), 1,2,3-benzenetriol sulfate, 1H-indole-7-acetic acid, 2-methoxyacetaminophen sulfate, 3-(3-hydroxyphenyl) propionate, 3-hydro-2-ethylpropionate, 3-methyl-2-oxovalerate, 3-methylhistidine, 4-ethylphenylsulfate, 4-guanidinobutanoate, 5-hydroxyhexanoate, 5-alpha-pregnan-3beta-ol,20-one sulfate, 7α-hydroxy-4-cholesten-3-one, acetic acid, acetyl-N-formyl-5-methoxykynurenamine, adrenoylcarnitine (C22:4), alliin, arginine, beta-cryptoxanthin, betaine, C4 carnitine, caproic acid, chenodeoxycholate, citric acid, citrulline, conjugated lineolic acid (18:2), D-proline, dihomo-linolysogycerol(20:3), dimethylglycine, ethyl propanoate, glutamine, glycocholic acid, glycodeoxycholic acid, glycolithocholic acid, GPC(18:0), ɣ-glutamylvaline, hyocholate, indoxyl sulfate, isovalerate, isovalerylcarnitine, kynurenine, kynurenine-tryptophan ratio, L-arginine, linoleoyl-arachidonoyl-glycerol (18:2/20:4), LPC (18:2), LPC (20:4), lysine, margarate, methylamine, MG(15:0), N-acetyl-cadaverine, N-acetylglutamine, N-linoleoylglycine, N-steroyl-sphinganine (d18:0/18:0), nicotinate beta-d-ribonucleotide, nonadecanoate, PE(p-16:0/20:4), perlolyrine, phenol sulfate, phenylacetic acid, phosphoniodidous acid, proline, prolylalanine, sebacate, taurodeoxycholic acid, taurolithocholate 3-sulfate, tauroursodeoxycholic acid, threonate, valerate. The amino acids: alanine, glutamate, histidine, leucine, tryptophan, tyrosine, and valine were each in two studies but with only very weak associations to individual microbe relative abundances.

| **Metabolite** | **Metabolite Class** | **Subclass** | **KEGG Class** | **KEGG Subclass** |
| --- | --- | --- | --- | --- |
| 3-phenylpropionate (hydrocinnamate) (3-PPA) | Aromatic Compound | Microbial Metabolite | Xenobiotics Biodegradation | Phenylpropanoid biosynthesis |
| 5-alpha-androstan-3beta,17alpha-diol disulfate | Steroids | Androgens | Steroids and Steroid Derivatives | Androgens and derivatives |
| Cinnamoylglycine | Aromatic Acid Derivative | Microbial-Mammalian Co-metabolite | Amino Acid Derivative | Phenylalanine metabolism |
| Deoxycholic acid (DCA) | Bile Acid | Secondary Bile Acid | Lipid | Secondary bile acid biosynthesis |
| Glycochenodeoxycholate (GCDCA) | Bile Acid Conjugate | Primary Bile Acid | Lipid | Primary bile acid biosynthesis |
| Glycochenodeoxycholate (GCDCA) sulfate | Bile Acid Conjugate | Sulfated Bile Acid | Lipid | Primary bile acid biosynthesis |
| Glycochenodeoxycholate (GCDCA) glucuronide | Bile Acid Conjugate | Glucuronide Conjugate | Lipid | Primary bile acid biosynthesis |
| Glycolithocholate (GLCA) | Bile Acid Conjugate | Glycine Conjugate | Lipid | Secondary bile acid biosynthesis |
| Glycolithocholate (GLCA) sulfate | Bile Acid Conjugate | Sulfated Bile Acid | Lipid | Secondary bile acid biosynthesis |
| Glycoursodeoxycholate (GUDCA) | Bile Acid Conjugate | Glycine Conjugate | Lipid | Secondary bile acid biosynthesis |
| Hippurate | Aromatic Compound | Microbial-Mammalian Co-metabolite | Amino Acid Derivative | Phenylalanine metabolism |
| Hyodeoxycholic acid | Bile Acid | Secondary Bile Acid | Lipid | Secondary bile acid biosynthesis |
| Imidazole propionate | Imidazole Derivative | Microbial Metabolite | Other Amino Acids | Histidine metabolism |
| Indole-3-propionate (I3P) | Indole Derivative | Microbial Metabolite | Other Amino Acids | Tryptophan metabolism |
| Isoursodeoxycholate (IUDCA) | Bile Acid | Secondary Bile Acid | Lipid | Secondary bile acid biosynthesis |
| Lithocholate (LCA) sulfate | Bile Acid Conjugate | Sulfated Bile Acid | Lipid | Secondary bile acid biosynthesis |
| p-Cresol glucuronide (PCG) | Phenol Derivative | Microbial-Mammalian Co-metabolite | Xenobiotics Biodegradation | Toluene degradation |
| p-Cresol sulfate (PCS) | Phenol Derivative | Microbial-Mammalian Co-metabolite | Xenobiotics Biodegradation | Toluene degradation |
| Phenylacetylglutamine (PAG) | Aromatic Compound | Microbial-Mammalian Co-metabolite | Amino Acid Derivative | Phenylalanine metabolism |
| Phenylalanine | Amino Acid | Aromatic Amino Acid | Amino Acid | Phenylalanine, tyrosine, and tryptophan metabolism |
| Trimethylamine N-oxide (TMAO) | Amine Oxide | Microbial Metabolite | Amino Acid metabolism | Choline/betaine metabolism |
| Ursodeoxycholate (UDCA) | Bile Acid | Secondary Bile Acid | Lipid | Secondary bile acid biosynthesis |

**Table S3 – Overview of Candidate Metabolites.** Metabolites selected from secondary studies comparing metabolites with microbiome

| **Metabolite** | **Study** | | | | | | | | | | | | | | | | | | | **Total** |
| --- | --- | --- | --- | --- | --- | --- | --- | --- | --- | --- | --- | --- | --- | --- | --- | --- | --- | --- | --- | --- |
|  | **1** | **2** | **3** | **5** | **6** | **7** | **8** | **9** | **10** | **11** | **12** | **13** | **15** | **16** | **18** | **19** | **20** | **21** | **22** |  |
| 3-Phenylpropionate |  |  |  |  |  |  |  |  |  |  |  |  |  |  |  |  |  |  |  | 10 |
| 5-alpha-androstan-3-beta-17-alpha-diol |  |  |  |  |  |  |  |  |  |  |  |  |  |  |  |  |  |  |  | 8 |
| Cinnamoylglycine |  |  |  |  |  |  |  |  |  |  |  |  |  |  |  |  |  |  |  | 7 |
| Deoxycholate |  |  |  |  |  |  |  |  |  |  |  |  |  |  |  |  |  |  |  | 12 |
| Glycochenodeoxycholate |  |  |  |  |  |  |  |  |  |  |  |  |  |  |  |  |  |  |  | 10 |
| Glycochenodeoxycholate glucuronide |  |  |  |  |  |  |  |  |  |  |  |  |  |  |  |  |  |  |  | 8 |
| Glycochenodeoxycholate sulfate |  |  |  |  |  |  |  |  |  |  |  |  |  |  |  |  |  |  |  | 8 |
| Glycolithocholate sulfate |  |  |  |  |  |  |  |  |  |  |  |  |  |  |  |  |  |  |  | 9 |
| Glycolithocholate |  |  |  |  |  |  |  |  |  |  |  |  |  |  |  |  |  |  |  | 4 |
| Glycoursodeoxycholate |  |  |  |  |  |  |  |  |  |  |  |  |  |  |  |  |  |  |  | 10 |
| Hippurate |  |  |  |  |  |  |  |  |  |  |  |  |  |  |  |  |  |  |  | 11 |
| Hyodeoxycholate |  |  |  |  |  |  |  |  |  |  |  |  |  |  |  |  |  |  |  | 1 |
| Imidazole propionate |  |  |  |  |  |  |  |  |  |  |  |  |  |  |  |  |  |  |  | 10 |
| Indole-3-propionate |  |  |  |  |  |  |  |  |  |  |  |  |  |  |  |  |  |  |  | 13 |
| Isoursodeoxycholate |  |  |  |  |  |  |  |  |  |  |  |  |  |  |  |  |  |  |  | 6 |
| Lithocholate sulfate |  |  |  |  |  |  |  |  |  |  |  |  |  |  |  |  |  |  |  | 6 |
| p-Cresol glucuronide |  |  |  |  |  |  |  |  |  |  |  |  |  |  |  |  |  |  |  | 9 |
| p-Cresol sulfate |  |  |  |  |  |  |  |  |  |  |  |  |  |  |  |  |  |  |  | 10 |
| Phenylacetylglutamine |  |  |  |  |  |  |  |  |  |  |  |  |  |  |  |  |  |  |  | 11 |
| Phenylalanine |  |  |  |  |  |  |  |  |  |  |  |  |  |  |  |  |  |  |  | 16 |
| Trimethylamine N-oxide |  |  |  |  |  |  |  |  |  |  |  |  |  |  |  |  |  |  |  | 10 |
| Ursodeoxycholate |  |  |  |  |  |  |  |  |  |  |  |  |  |  |  |  |  |  |  | 7 |
| **Total** | 1 | 2 | 21 | 0 | 7 | 12 | 21 | 21 | 3 | 0 | 6 | 0 | 1 | 18 | 20 | 19 | 13 | 12 | 19 |  |

**Table S4** – Visualization of different candidate metabolites represented in studies included in analysis. For study numbers see Table S5 (following).

| **Study** | **Author** | **N (complete)** | **Inclusion/Exclusion** | **Candidate Metabolites** | **Total Metabolites** | **Conventional Meta-Analysis** | **Patient Level Meta-Analysis** | **Case-Control** | **Longitudinal Analysis** |
| --- | --- | --- | --- | --- | --- | --- | --- | --- | --- |
| 1 | Wang | 40 (35) | Included | 1 | 80 | No | Yes | No | No |
| 2 | Bishehsari | 123 (118) | Included | 2 | 23 | No | Yes | No | No |
| 3 | Zeybel | 41 (41) | Included | 21 | 1032 | No | Yes | Yes | No |
| 4 | Wei | 183 (NA) | Microbiome files missing | 3 | 5 | No | No | No | No |
| 5 | Lin | 517 (498) | Included | 0 | 6 | No | No | No | No |
| 6 | Schirmer 2016 | 488 (441) | Included | 7 | 458 | Yes | Yes | No | No |
| 7 | Chen | 45 (45) | Included | 12 | 5514 | Yes | Yes | Yes | No |
| 8 | Fulcher | 28 (28) | Included | 21 | 1201 | Yes | Yes | Yes | No |
| 9 | Fromentin | 1087 (992) | Included | 21 | 1513 | Yes | Yes | Yes | No |
| 10 | Tanes | 30 (30) | Included | 3 | 56 | Yes | Yes | No | Yes |
| 11 | Sereti | 137 (130) | Included | 0 | 8 | No | No | No | No |
| 12 | Ma | 59 (58) | Included | 6 | 388 | Yes | Yes | Yes | No |
| 13 | Uema | 140 (140) | Included | 0 | 57 | No | No | No | No |
| 14 | Xu | 383 (NA) | Unable to match files | 21 | 821 | No | No | No | No |
| 15 | Riwes | 25 (25) | Included | 1 | 90 | No | Yes | No | No |
| 16 | Gershuni | 48 (44) | Included | 18 | 825 | No | Yes | No | No |
| 17 | Louca | 3754 (NA) | Unable to match files | 8 | 19 |  |  |  |  |
| 18 | Shoer | 152 (148) | Included | 20 | 1095 | Yes | Yes | No | No |
| 19 | Rashidi | 46 (34) | Included | 19 | 945 | Yes | Yes | No | Yes |
| 20 | Schirmer 2024 | 241 (85) | Included | 13 | 10,026 | Yes | Yes | No | No |
| 21 | Levi | 94 (68) | Included | 12 | 518 | Yes | Yes | No | No |
| 22 | Kang | 38 (38) | Included | 19 | 888 | No | Yes | No | No |
| 23 | Kordy | 80 (68) | Composition differed from original paper | 20 | 846 | No | No | No | No |
| 24 | Gao | 100 (NA) | Unable to match files | 21 | 792 | No | No | No | No |

**Table S5** – overview of all studies in review.

| **Category** | **Metabolites** | **Direction of Change Expected with Anaerobe Loss** | **Notes** |
| --- | --- | --- | --- |
| Strict Anaerobe-Dependent Products | 3-PPA, Cinnamoylglycine, DCA, Hippurate, Hyodeoxycholate, Indole-3-propionate, IUDCA, LCA, UDCA |  | Healthy microbial axis |
| Production dependent on host metabolism | GLCA, GLCA sulfate, GUDCA | / | Dependent on deconjugation capacity |
| Some facultative anaerobe redundant metabolism | Imidazole propionate | / | Context dependent |
| Substrates | GCDCA, GCDCA sulfate, GCDCA glucuronide, Phenylalanine | / | Intake and host metabolism dependent |
| Facultative anaerobe/pathogen-associated | 5-alpha-androstane-3-beta-17-alpha-diol, PAG, p-cresol glucuronide, p-cresol sulfate, TMAO |  | “Dysbiosis” or overflow axis |

**Table S6 –** Predicted direction of change in metabolites based on microbial activity. DCA=Deoxycholate, GLCA=Glycolithocholate, LCA=Lithocholate, UDCA=Ursodeoxycholate, IUDCA=Isoursodeoxycholate, GUDCA=Glycoursodeoxycholate, 3-PPA=3-Phenylpropionate; PAG=Phenylacetylglutamine, GCDCA=Glycochenodeoxycholate, TMAO=Trimethylamine-N-oxide

| Study Author | Study Size (n) | Variance | Std Dev | Median | IQR |
| --- | --- | --- | --- | --- | --- |
| Kang | 38 | 0.0007 | 0.0261 | 0.969 | 0.0369 |
| Sereti | 130 | 0.0014 | 0.0369 | 0.964 | 0.0280 |
| Wang | 35 | 0.0015 | 0.0388 | 0.955 | 0.0410 |
| Bishehsari | 118 | 0.0017 | 0.0416 | 0.964 | 0.0338 |
| Zeybel | 41 | 0.0025 | 0.0497 | 0.990 | 0.0146 |
| Fromentin | 994 | 0.0030 | 0.0546 | 0.955 | 0.0488 |
| Lin | 499 | 0.0047 | 0.0685 | 0.927 | 0.0567 |
| Levi | 94 | 0.0100 | 0.1001 | 0.845 | 0.1374 |
| Schirmer 2016 | 471 | 0.0127 | 0.1128 | 0.893 | 0.1349 |
| Gershuni | 45 | 0.0128 | 0.1133 | 0.988 | 0.0306 |
| Uema | 140 | 0.0133 | 0.1155 | 0.865 | 0.1347 |
| Chen | 45 | 0.0330 | 0.1819 | 0.933 | 0.1614 |
| Shoer | 152 | 0.0331 | 0.1819 | 0.808 | 0.3028 |
| Riwes | 25 | 0.0419 | 0.2046 | 0.990 | 0.0604 |
| Schirmer 2024 | 241 | 0.0455 | 0.2132 | 0.936 | 0.1350 |
| Fulcher | 28 | 0.0467 | 0.2160 | 0.893 | 0.2523 |
| Ma | 59 | 0.1053 | 0.3245 | 0.642 | 0.5351 |
| Rashidi | 46 | 0.1262 | 0.3553 | 0.766 | 0.6306 |
| Tanes | 30 | 0.1264 | 0.3555 | 0.821 | 0.6104 |

**Table S7 –** Distribution of anaerobe relative abundance (RA) across included studies ordered by variance.

| **Microbiome** | | | |
| --- | --- | --- | --- |
| **Study** | **Predicted** | **Actual** | |
|  |  | Case | Control |
| Fromentin (2022) | Case | 211 | 81 |
|  | Control | 1 | 0 |
| Fulcher (2022) | Case | 0 | 1 |
|  | Control | 2 | 5 |
| Ma (2021) | Case | 8 | 0 |
|  | Control | 0 | 9 |
| Chen (2022) | Case | 9 | 3 |
|  | Control | 0 | 1 |
| Zeybel (2022) | Case | 9 | 2 |
|  | Control | 0 | 0 |
| **Metabolites** | | | |
| Fromentin (2022) | Case | 198 | 54 |
|  | Control | 14 | 27 |
| Fulcher (2022) | Case | 6 | 2 |
|  | Control | 0 | 0 |
| Ma (2021) | Case | 8 | 0 |
|  | Control | 0 | 9 |
| Chen (2022) | Case | 6 | 4 |
|  | Control | 3 | 0 |
| Zeybel (2022) | Case | 9 | 2 |
|  | Control | 0 | 0 |

**Table S8 –** Microbiome and Metabolite random forest classifier case-control prediction**.**

| DEPOSITED DATA | SOURCE | IDENTIFIER |
| --- | --- | --- |
| Wang et al. (2017) | SRA | SRP103896 |
| Bishehsari et al. (2023) | SRA | SRP133159 |
| Zeybel et al. (2022) | Original paper | Dataset S3; https://advanced.onlinelibrary.wiley.com/doi/full/10.1002/advs.202104373 |
| Lin et al. (2023) | SRA | PRJNA986283 and PRJNA1011937 |
| Schirmer et al. (2016) | SRA | PRJNA319574 |
| Chen et al. (2022) | Original paper; Mendeley data | https://data.mendeley.com/datasets/8rftx9ybnm/1 |
| Fulcher et al. (2022) | SRA | PRJNA836336 |
| Fromentin et al. (2022) | Original paper | Supplementary table 10; https://www.nature.com/articles/s41591-022-01688-4 |
| Tanes et al. (2021) | SRA | PRJNA675301 |
| Sereti et al. (2023) | SRA | PRJNA994573 |
| Ma et al. (2021) | Original paper | https://pubs.rsc.org/en/content/articlelanding/2021/mo/d1mo00279a |
| Uema et al. (2022) | Original paper; Mendeley data | https://data.mendeley.com/datasets/z483h65xrp/2 |
| Riwes et al. (2023) | SRA | PRJNA953087 |
| Gershuni et al. (2021) | Original paper; Github | https://github.com/yli0131/MGM |
| Shoer et al. (2023) | ENA | PRJEB64861 |
| Rashidi et al. (2022) | SRA | SRP141394 |
| Schirmer et al. (2024) | Original paper | Table S4; https://www.sciencedirect.com/science/article/pii/S1931312823005085 |
| Levi et al. (2021) | Original paper | Table S3; https://www.sciencedirect.com/science/article/pii/S2666379121000628?via%3Dihub |
| Kang et al. (2020) | SRA | PRJNA529598 |

**Table S9 –** Key resources table for original locations of all primary data (microbiome and metabolite data)**.**
